# Neuartige Variante des *NCSTN*‐Gens bei einer Frau mit familiärer Hidradenitis suppurativa

**DOI:** 10.1111/ddg.15792_g

**Published:** 2025-10-23

**Authors:** Conrad Hempel, Sonja Grunewald, Till Mittank‐Weidner, Jan‐Christoph Simon, Franziska Schnabel, Robin‐Tobias Jauss, Viktor Schnabel

**Affiliations:** ^1^ Klinik und Poliklinik für Dermatologie Venerologie und Allergologie Universitätsklinikum Leipzig; ^2^ Institut für Humangenetik Universitätsklinikum Leipzig

**Keywords:** familiäre Akne inversa, Hidradenitis suppurativa, *NCSTN*‐Genvariante, familiar acne inversa, Hidradenitis suppurativa, *NCSTN* gene mutation

Sehr geehrte Herausgeber,

Eine 27‐jährige kaukasische Frau mit einer vierjährigen Vorgeschichte einer Hidradenitis suppurativa stellte sich in unserer Ambulanz vor. Sie berichtete von wiederholten umfangreichen chirurgischen Eingriffen mit Spaltung von Hautabszessen in der Achselhöhle und in der Leiste, die interessanterweise auch an ungewöhnlichen Stellen wie Rumpf und Oberschenkeln auftraten. Zuvor wurde sie mit intermittierenden Antibiotika, Isotretinoin und einem hormonellen Kontrazeptivum behandelt. Zusätzlich wurde eine Therapie mit Tumornekrosefaktor‐alpha‐Antikörpern für 6 Monate sowie zuletzt eine Anti‐IL17‐Therapie für 3 Monate durchgeführt. Alle Medikamente sind aufgrund von Nebenwirkungen oder Unwirksamkeit abgesetzt worden.

Sie klagte vor allem über starke Schmerzen infolge der wiederkehrenden Abszesse, die zu einer Arbeitsunfähigkeit führten. Sie verneinte Nikotinkonsum und hat einen BMI von 18,5. Aufgrund einer begleitenden Depression und Angststörung befindet sie sich in psychologischer Betreuung und hat einen DLQI‐Wert von 29.

Die dermatologische Untersuchung ergab zahlreiche entzündliche Papeln, Komedonen und Pusteln, die über den ganzen Körper verteilt waren, wobei Gesicht, Rumpf und Oberschenkel am stärksten betroffen waren. Abszesse mit einem Durchmesser von bis zu 2 cm fanden sich in beiden Achselhöhlen sowie im Scham‐ und Gesäßbereich (Abbildung 1a,b), entsprechend einem Hurley‐Grad 2. Interessanterweise wiesen ihre Mutter und ihr Onkel mütterlicherseits ebenfalls intermittierende schmerzhafte Abszesse auf, die jedoch weniger stark ausgeprägt waren (Abbildung S1, Online‐Supplement).

**ABBILDUNG 1:**
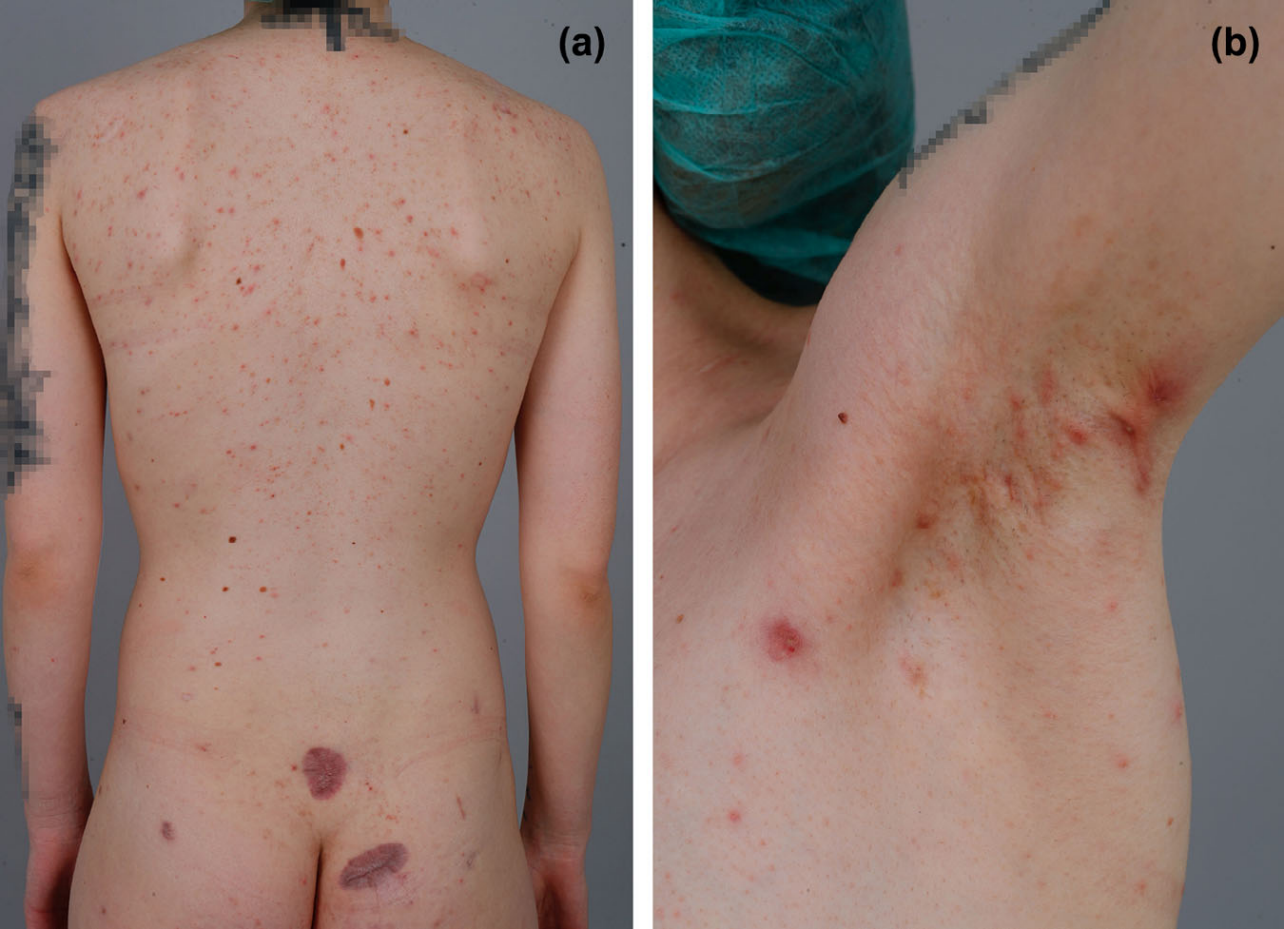
(a) Rücken und (b) linke Achselhöhle einer 27‐jährigen Frau mit familiärer HS, die durch eine *NCSTN*‐Variante verursacht wird, mit multiplen entzündeten Papeln und Komedonen, die über den gesamten Rumpf verteilt sind. Zwei flache Narben auf dem unteren Rücken/Gesäß von früheren Abszessexzisionen.

Aufgrund der positiven Familienanamnese führten wir eine Whole‐Exom‐Sequenzierung mit dem TWIST Human Core Exome Kit (TWIST Bioscience, San Francisco, USA) auf einem Illumina NovaSeq6000‐Sequenzierer (Illumina, San Diego, USA) durch. Die Auswertung erfolgte mit der browserbasierten Genomiksoftware Varvis (Limbus Medical Technologies GmbH, Rostock, Deutschland). Hierbei zeigte sich eine heterozygote trunkierende Variante (NM_015331.3:c.1101_1101+17delinsTGTCCA, p.(Gln367Hisfs*6)) im *NCSTN*‐Gen (Abbildung S2, Online‐Supplement). Die mittlere Abdeckung des *NCSTN*‐Gens betrug 79,49x, und die Variante wurde in mehr als 50 Reads mit einer Qualitätsbewertung von 1918 (GATK HaplotypeCaller) und ohne diskrepante Reads in diesem Bereich nachgewiesen. Zur Bestätigung wurde eine Sanger‐Sequenzierung durchgeführt (Abbildung S3, Online‐Supplement). Die Variante führt zu einem Frameshift mit verkürzter Proteintranslation, wurde bislang jedoch weder in Varianten‐Datenbanken (HGMD, Decipher, ClinVar) noch in der Literatur beschrieben. In der allgemeinen Referenzdatenbank für Populationsfrequenzen (gnomAD) die Sequenzdaten von mehr als 1 150 000 europäischen (nicht finnischen) Kontrollpersonen an der Variantenposition enthält, ist die Variante nicht gelistet. Nach der ACMG‐Klassifikation wurde die Variante als pathogen eingestuft (angewandte Kriterien: PVS1, PM2_SUP, PP4).^1^ Leider standen keine weiteren Familienmitglieder für eine Segregationsanalyse zur Verfügung.

Hidradenitis suppurativa (HS), auch bekannt als Acne inversa, ist eine chronische, entzündliche und rezidivierende Erkrankung der Haarfollikel, die sich typischerweise durch schmerzhafte, entzündete Läsionen äußert. Am häufigsten sind die axilläre, inguinale und anogenitale Region betroffen.^2^ Die Prävalenz in Europa liegt bei etwa 1%.^2^ Das typische Erkrankungsalter bei Erstmanifestation liegt zwischen 20 und 40 Jahren.^3^ Etwa 40% der Fälle lassen sich als familiäre Form einordnen, neben syndromalen und sporadischen Varianten.^3^ Die familiäre HS ist in der Regel durch schwerere Symptome und einen früheren Krankheitsbeginn gekennzeichnet.^3^ In der Literatur wurden bisher mindestens vier verschiedene Gene aus dem y‐Sekretase‐Komplex als ursächlich für HS beschrieben, darunter *NCSTN, PSENEN, PSEN1 und APH1B*.^4^, ^5^ *NCSTN* kodiert für Nicastrin, ein Typ‐1‐Transmembran‐Glykoprotein des y‐Sekretase‐Komplexes, das eine zentrale Rolle im Notch‐Signalweg spielt und antiproliferative und differenzierungsfördernde Effekte in menschlichen Keratinozyten steuert. Die Ausschaltung von *NCSTN* führt zu einer verstärkten Proliferation und verminderten Differenzierung von Keratinozyten, die in erster Linie durch den Notch‐ und PIK‐AKT‐Signalweg vermittelt wird.^6^ Bisher wurden 52 Varianten von *NCSTN* bei HS beschrieben.^5^ Eine monogene Variante von *NCSTN* ist mit einem früheren Auftreten und einer atypischen Präsentation an ungewöhnlichen Hautstellen wie dem Rumpf verbunden. Darüber hinaus unterscheiden ein niedriges Körpergewicht und eine über den Krankheitsverlauf erhöhte Wahrscheinlichkeit einer Behandlung mit biologischen Therapien diese genetische Varianten von der klassischen HS, wie dieser Fall bestätigt.^7^ Das Zusammenspiel zwischen genetischen Faktoren und der Dysregulation von Immunmediatoren wie TNF‐α, IL‐1β, IL‐17 und IL‐12/23 trägt zum chronisch‐entzündlichen Charakter der HS bei.^3^ Potenzielle therapeutische Ziele umfassen daher Anti‐TNF‐α‐, Anti‐IL‐1α‐, Anti‐IL‐17‐ und Anti‐IL‐12/23‐Therapien sowie JAK‐Inhibitoren.

Dieser Fall untermauert die funktionelle Bedeutung von *NCSTN*‐Varianten in der Pathogenese der familiären HS, die bisher vor allem in asiatischen Populationen beschrieben wurde.^8^, ^9^

Bei unserer Patientin haben wir eine neue trunkierende *NCSTN*‐Variante beschrieben, die zur Entwicklung von HS führt. Interessanterweise konnten keine umweltbedingten Risikofaktoren, wie Adipositas oder Rauchen, festgestellt werden. Klinisch gehört der Phänotyp zum selteneren „follikulären“ Subtyp der HS, der von Canoui‐Poitrine et al. als einer von drei Subtypen beschrieben wurde.^10^ Dieser ist hauptsächlich durch follikuläre Läsionen wie Komedonen, epidermale Zysten sowie dem Auftreten von Sinus pilonidalis und Akne vulgaris gekennzeichnet und zeigt einen schwereren Krankheitsverlauf als die reguläre axilläre/inguinale Variante. Es muss darauf hingewiesen werden, dass die Komedonen unserer Patientin nicht die alleinige Ursache für die schwere, destruktive Entzündung sein können. Angesichts des genetischen Hintergrunds liefert dieser Bericht weitere Hinweise darauf, dass HS nicht länger als einfache „Erkrankung der Follikelokklusion“ betrachtet werden kann. Die bedeutende Rolle der Entzündung sowie der angeborenen und adaptiven Immunität bei familiärer HS kann nicht hoch genug eingeschätzt werden und wirft die Frage auf, ob die Follikelokklusion ein primäres oder sekundäres Phänomen in der Pathogenese der Erkrankung ist.

Unser Fallbericht erweitert das genetische Spektrum, das der HS zugrunde liegt. Zusammen mit dem klinischen Phänotyp liefert er wertvolle Informationen für weitere Studien zur Therapie und zur Entwicklung von Überwachungsprogrammen für betroffene Familien.

## DANKSAGUNG

Open access Veröffentlichung ermöglicht und organisiert durch Projekt DEAL.

## INTERESSENKONFLIKT

Keiner.

## Supporting information



Supplementary information

Supplementary information

Supplementary information
